# Two Novel Compounds with Tri-aryl Structures as Effective Anti-Breast Cancer Candidates *In-vivo*

**DOI:** 10.22037/ijpr.2019.111802.13366

**Published:** 2020

**Authors:** Ahoo Afsharinasab, Fariborz Moayer, Mohsen Amini, Samira Choopani, Raheleh Tahmasvand, Soudeh dehghani, Seyede Zahra Mousavi, Mona Salimi

**Affiliations:** a *Department of Pharmacology and Toxicology, Faculty of Pharmacy and Pharmaceutical Sciences, Tehran Medical Sciences, Islamic Azad University, Tehran, Iran (IAUPS). *; b *Department of Physiology and pharmacology, Pasteur Institute of Iran, Tehran, Iran. *; c *Department of Pathobiology, College of Veterinary Medicine, Karaj Branch, Islamic Azad University, Alborz, Iran. *; d *Department of Medicinal Chemistry, Faculty of Pharmacy, Tehran University of Medical Sciences, Tehran, Iran.*

**Keywords:** Breast cancer, BALB/c, Tri-aryl structure, 4T1cell, Histopathology

## Abstract

Prognosis of metastatic breast cancer is very poor which urges the necessity to develop novel potential drug candidates. We assessed two compounds with tri-aryl structures (**A** and **B**) for their potency to reduce primary breast tumor growth and lung metastasis in 4T1 mice model. MTT assay, 4T1 mammary mouse model, and immunohistochemistry experiments were used in this study. *In-vitro* results exhibited an anti–proliferative effect for compounds **A** and **B** towards MDA-MB-231 cancer cells. Our *in-vivo* results displayed that administered compounds **A** and **B** could suppress the size of the primary tumor and the number of lung metastatic foci in 4T1 BALB/c mice model. Histopathological analysis revealed that treatment of both compounds resulted in necrosis. Our findings provide new evidence that compound **B** may be promising for slowing the growth of tumor along with metastatic foci via COX-2 independent pathway.

## Introduction

Breast cancer is considered as the most familiar female malignant tumor in western countries and is becoming more and more widespread in Asia (1). Currently, the survival of patients with metastatic breast cancer remains low at only 23% (2, 3). This issue prompted researchers to make a mouse model to study breast cancer and its progression as well as develop novel and more effective compounds (4, 5). Among the different models, an excellent currently available model of breast cancer is the BALB/c-derived 4T1 tumor. This tumor model shares many features with human breast cancer in terms of progressive growth in the mammary gland and active metastasis to the other organs (6).

Chemotherapy is unable to obtain clinical responses in patients with highly invasive metastatic disease (7-9). Several limitations are reported with chemotherapy using, among them, tumor drug resistance and risk of toxicities are the most important ones (10-12). Therefore, there is an essential need for more effective approaches to treatment of breast cancer (13, 14). Nonsteroidal anti-inflammatory drugs (NSAIDs) have the potential to be used as anti-cancer agents (15). In this regard, celecoxib as the first selective cyclooxygenase-2 (COX-2) inhibitor has been approved for treatment of different types of cancer, which acts through COX-2-dependent and -independent mechanism (11, 16).

**Figure 1:**
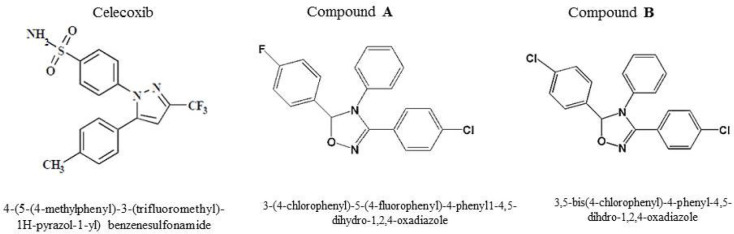
Chemical structures of compounds **A** and **B**

Celecoxib with a 1, 2-di-aryl heterocyclic structure is an ideal lead compound for developing novel derivatives possessing potent anticancer property (17). Currently, researchers directed more attention towards compounds with tri-aryl structures as more potent chemotherapeutic or chemo-preventive COX-2 inhibitor agents (18). We have recently reported that two tri-aryl structure compounds (**A, B**) (Figure 1) displayed a significant anti-proliferative activity with considerable IC_50_ values (6.5 and 10.1 µM) on breast adenocarcinoma (MCF-7) cell line after 24 h of treatment (17). Considering these data and knowing that triple negative breast cancer is one of the most complicated subtypes of breast cancers with high aggressiveness and poor prognosis, we decided herein to evaluate the antitumor property of compounds **A** and **B** in both *in-vitro* and i*n-vivo *models resembling the human triple negative breast cancer.

## Experimental

*Chemicals and cells*

Human breast adenocarcinoma (MDA-MB-231, C578) and mouse mammary tumor (4T1, C604) cell lines were purchased from National Cell Bank of Pasteur Institute of Iran (NCBI). The cells were cultured in Dulbecco’s modified Eagle’s medium (DMEM) (Gibco-BRL, Rockville, IN) containing 10% fetal bovine serum (FBS) (Gibco-BRL, Rockville, IN), and 1% Penicillin/Streptomycin (Gibco-BRL, Rockville, IN). Compounds **A** and **B** were synthesized in the medicinal chemistry laboratory at the faculty of pharmacy of Tehran University of Medical Sciences. Celecoxib was kindly provided by Pars Daru (Tehran, Iran). All other chemicals were in high purity and prepared from Merck (Darmstadt, Germany) and Sigma–Aldrich (StLouis, MO).

*MTT assay*

MTT assay was employed to assess the inhibitory effect of compounds **A **and **B **on the cell growth. To do it, MDA-MB-231 cells (5×10^3^ cells/well) were seeded in 96-well plates and incubated at 37 °C in a humidified 5% CO2 incubator. Then, the cells were treated with various concentrations (0.1-100 µM) for 72 h. Untreated cells as well as 0.3% DMSO- treated cells served as negative and vehicle controls. Following addition 20 µL of (3-(4,5-dimethylthiazol-2-yl)-2,5-diphenyl tetrazolium bromide (MTT, 5 mg/mL), cells were further incubated at 37 °C for 4 h. The supernatants were then aspirated, and 200 µL of dimethylsulfoxide (DMSO) were used to dissolve purple formazan in each well. The plates were shaken for another 15 min and the absorbance was read using a Microplate Reader (Star Fax-2100, ST. Louis, USA) at 545 nm. The percentage of cytotoxicity was determined using the following formula.

% of cell cytotoxicity

100-[(Abs (drug)/ Abs (control)*100]

*Animals*

A total of 98 female 6-8 weeks-old BALB/c mice used in our study were prepared from the National Animal Center (Pasteur Institute of Karaj) and maintained in a 12/12-h light–dark cycle, with food and water supplied *ad libitum*. The animals were treated in accordance with the guidelines approved by the animal ethics committee of Pasteur Institute of Iran. After that, exponentially 4T1 cells were trypsinized and 10^6 ^cells were re-suspended in PBS and inoculated into the mammary fat pad of the mice. Simultaneously, compounds **A** and **B** at doses of 1, 5, 10, and 15 mg/kg/day were i.p. administered five times a week for four weeks. Animal weight and tumor volume were measured once per week. The tumor volumes (mm^3^) were calculated in two dimensions using a digital caliper through the following formula: (length×width^2^)/2.

*Histopathology*

For histopathological analysis, primary tumors and lungs, the major metastatic organ, were collected. Tumors and organs were removed at day 28 post-tumor injection and fixed in 10% buffered formalin for at least 24 h and then processed for routine paraffin embedding. Five-micron sections of each lesion were stained with hematoxylin–eosin (H&E). 

*Immunohistochemistry*

Immuno-histochemical assay was carried out on representative blocks of tumor tissues dissected from the mice treated with 10 and 15 mg/kg of compound **B**. Paraffin-embedded tumor sections (4 µm) were deparaffinized in xylene and rehydrated by graded alcohol and then incubated with anti- COX-2 antibody (RCM 306A, Biocare) followed by biotinylated secondary antibody using an HRP/DAB detection IHC kit (ab64264, Abcam, Cambridge, MA 02139-1517 ,UK) according to the manufacturer’s instructions and finally analyzed by an expert pathologist. Suitable positive control was run with each experiment.

*Statistical analysis*

The presented data are mean ± SEM of at least triplicate determinations, and the comparisons were based on ANOVA followed by the Tukey’s post test using GraphPad Prism software version 6. A *p* value lower than 0.05 was considered as significant.

## Results

*Anti-proliferative activity of compounds* ***A*** *and* ***B***

Anti-proliferative effects of compounds **A** and **B** were assessed by using MTT assay against invasive human breast cancer cell (MDA-MB-231) and the IC_50_ values were reported in (Table 1). The results revealed a potent anti-proliferative effect against cancer cells for compound **B** after 72 h of incubation with the IC_50_ value of 9.2 µM. Considering the IC_50_ values in (Table 1), compound **B** was superior to compound **A** in inhibiting the invasive and proliferative cells (9.2 *vs.* 17.95 µM ). The highest anti-proliferative activity was for celecoxib displaying an IC_50_ value of 7.45 µM. 

*In-vivo inhibition efficiency*

**Figure 2:**
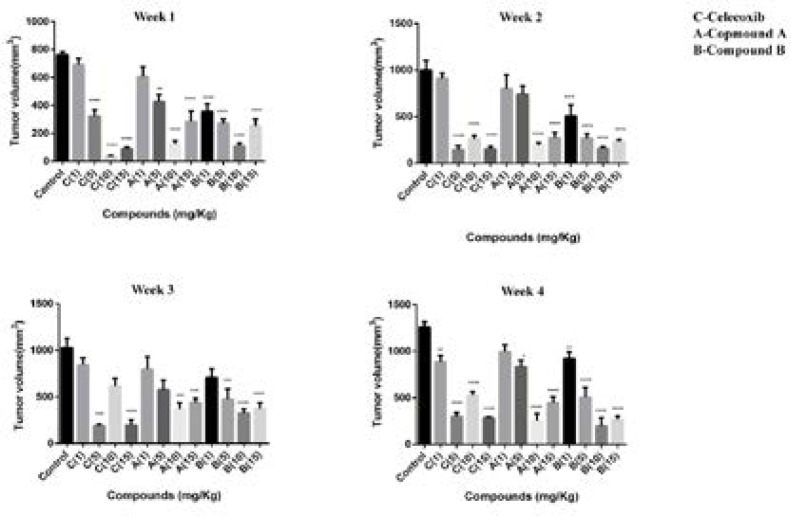
Tumor volume (mm3) in BALB/c mice with mammary cancer (4T1 breast cancer model) treated by celecoxib, compounds **A** and **B** at doses of 1,5,10 and 15 mg/kg/day for 4 weeks. One-way ANOVA test (post-Tukey test) done for n = 7 mice per group. Error bar indicates SEM, (^**^p < 0.01, ^***^p < 0.001, ^****^p < 0.0001 compared to the control group).

In our previous study, we reported firstly that two compounds (**A** and **B**) with tri-aryl structures displayed anti-breast cancer activity by the mechanism of cell apoptosis induction through a COX-2-independent pathway (17). The current work further confirmed our previous results in an *in-vivo* model. Hence, a 4T1 mammary carcinoma model was used to explore the potential properties of compounds **A** and **B** on breast cancer growth and metastasis. In this model, 4T1 cells were inoculated into fat mammary pad of BALB/c mice. Then, the mice were divided into 14 groups and treated with celecoxib, compounds **A**, and **B**. The i.p. administration was performed at an interval of 7 days for 5 times at doses of 1, 5, 10, and 15 mg/kg/day for 4 weeks. As depicted in Figure 2, administration of compounds **A** and **B** as well as celecoxib at doses of 1,5,10,15 mg/kg/day resulted in primary tumor size regression within the four weeks treatment; however, tumor size reduction was the most significant at week 4. 

**Figure 3:**
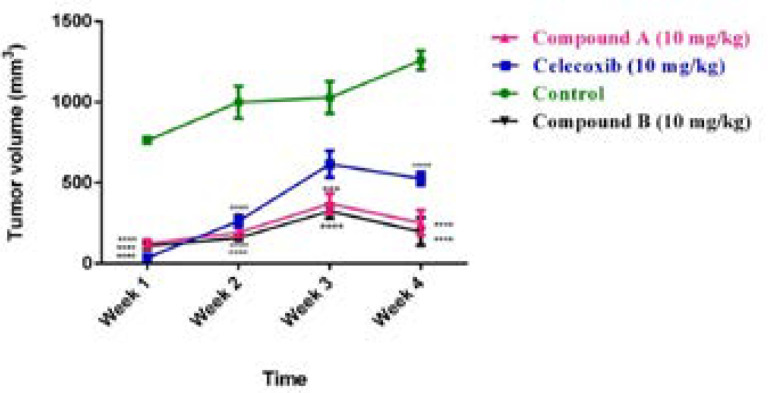
Comparing the effect of compounds **A** and **B** as well as celecoxib at dose of 10 mg/kg/day on tumor growth during the 4 weeks treatment. Data are expressed as mean ± SEM, n = 7 mice per group, ^***^p < 0.001, ^****^p < 0.0001 compared to the control group).

Our results also demonstrated that after treating for 28 days, the average tumor volume of the control group was about 1250 mm^3^. However, tumor size in mice was significantly reduced with treatment of both compounds and celecoxib. The mice treated with compound **A** had the average tumor volumes about 990, 830, 250, and 440 mm^3 ^at doses of 1,5,10,15 mg/kg/day, respectively, after 4 weeks of administration. The compound**-B** treated mice displayed better inhibition effect on tumor volume than compound **A**. Our results indicated that tumor sizes in the mice treated with 1, 5, 10, 15 mg/kg/day of compound **B **were 920, 505, 197, and 268 mm^3 ^at week 4. For celecoxib, tumor volumes were as follows at the mentioned doses after 4 weeks of treatment; 887, 300, 526, and 284 mm^3 ^(Figure 3). 

Our findings demonstrated that lower doses of compounds **A** and **B **(1 and 5 mg/kg/day) were unable to remarkably block the tumor progression; however, the animals treated with 10 mg/kg/day of both compounds displayed a more notable tumor size reduction compared with those treated with 1 and 5 mg/kg/day suggesting a dose-dependent anti-mammary tumor effect of the compounds. Interestingly, both compounds followed the same trend in the 4 -week treatment period and the most effective response was seen at the end of the study after 20 days of administration. In addition, celecoxib diminished the tumor size at different concentrations; however, among the various concentrations administered, 5 mg/kg/day of celecoxib was enough effective to reduce the tumor volume within the last three weeks of treatment, which may be attributed to the high potency of celecoxib compared with the two compounds. Interestingly, with the increasing dose of celecoxib to 10 mg/kg/day, its potential to repress the tumor growth was reduced. These findings suggest a negative feedback mechanism for celecoxib, which is line with a study performed by Ramer *et al*.(19). Noteworthy is mentioning that 10 mg/kg of daily administration of celecoxib was more effective than 5 mg/kg at the first week of treatment in tumor reduction size implying a time-dependent uptake efficiency for celecoxib. Based on our results obtained herein, we selected the dose of 10 mg/kg/day of the compounds to be administered by which the tumor size reduction was utmost and we subsequently carried out our further experiments at this dose. Surprisingly, with increasing the administered dose to 15 mg/kg/day, no effective response by tumor size reduction was observed in the mice treated with compounds **A **and **B**.

Moreover, no obvious indication of morphological change as well as weight loss was observed in the mice treated with compounds.

*Compounds* ***A*** *and* ***B*** *enhanced tumor necrosis*

**Figure 4:**
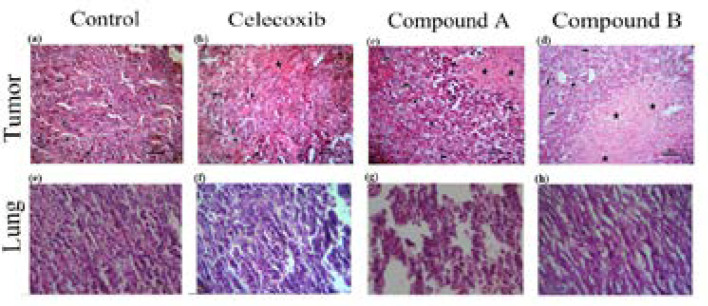
Effect of celecoxib and compounds **A** and **B **at dose of 10 mg/kg on solid tumors and lungs in Balb/c mice injected with 4T1 cells. The mice were killed 28 days after cell injection, tumor (a-d) and lung (e-h) sections were evaluated by H&E staining (original magnification ×200). *Small arrow:* inflammatory cells; *Big arrow:* tumor cells; *Star:* necrotic area; *Headache:* mitotic division

To confirm our *in- vivo* data and show whether compounds **A** and **B** demonstrate anti-tumor activity, histopathology studies were performed on tumor sections upon sacrificing mice on day 28. Based on the H&E staining results, as illustrated in (Figure 4) (a-d), the control group was composed of the enormous neoplastic epithelial cells possessing large round to oval nuclei and small to moderate amounts of eosinophilic cytoplasm. Also, a number of mitotic cells were remarkably observed in the tumor sections of control group. However, the sections of the tumor masses from the treatment groups, either compounds **A** and **B** or celecoxib, displayed differences in degree of necrosis; i.e. the lesion from the compound **B**-treated group revealed a marked necrosis within the tumor mass with less inflammatory cells at the periphery of the lesion than that of control and compound **A** treated-group at the same dose administered (10 mg/kg/day). Interestingly, the observed necrosis was even more than that of celecoxib-treated group

*Effect of the compounds on lung metastasis*

A hallmark of the malignant tumors is metastasis (21, 22). Thus, herein, we evaluated the anti-metastatic effect of the two compounds in the 4T1 tumor-bearing mouse model, which closely mimics metastatic breast cancer in human (23). 4T1 cells are approved to be highly invasive and primary cells usually metastasize to the lung following establishment for 2 to 3 weeks in BALB/c mice (23). In the current study, we investigated the effect of compounds **A** and **B** on the metastasis of breast cancer using 4T1 mammary carcinoma cells, which indicates a highly tumorigenic and invasive feature. To do it, the mice were killed and the lungs were subsequently dissected on the 28^th^ day following 4T1 cells inoculated (Figure 4 e-h). As shown in (Figure 4,e-h), compounds **A** and **B** suppressed the metastatic potential of 4T1 tumor cells. Metastasis incidence was seen in vehicle group, while the presence of metastatic cells in the pulmonary of compound-**B** treated group was significantly less than that of the compound **A-**treated group at the same dose (10 mg/kg/dose). 10 mg/kg/day of celecoxib treatment did not drastically diminish the number and size of lung metastasis nodules. This is the first report demonstrating that both compounds are effective not only in controlling the primary tumor size, but also in suppressing secondary lung metastasis.

*The association between expression of COX2 and tumor size*

**Figure 5.**
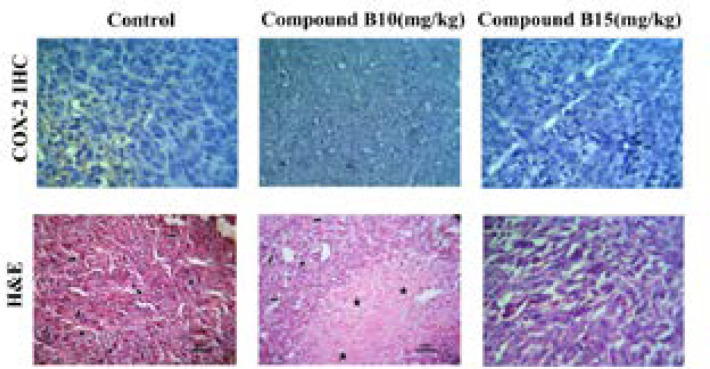
Expression of COX-2 in tumor tissues. Tumor tissues were collected from the mice receiving 10 and 15 mg/kg /day of compound B. Representative images of the immunohistochemical analysis are shown. All photomicrographs are at ×200 magnification.* Small arrow:* inflammatory cells; *Big arrow:* tumor cells; *Star:* necrotic area; *Headache:* mitotic division

According to the results of our *in-vivo* experiment, compound **B** at dose of 10 mg/kg/day was more effective than that of 15 mg/kg/day, in terms of tumor reduction size. In order to verify whether this effect may be due to an increase in COX-2 expression, IHC was applied to detect COX-2 immunopositive cells in the cytoplasm (Figure 5). The findings exhibited that COX-2 protein was weakly expressed in the tumors dissected from the control mice after 28 days. However, compound **B **at the both doses administered could inhibit COX-2 expression suggesting no association between COX-2 expression and tumor reduction size in compound **B** treated group at doses of 10 and 15 mg/kg/day.

These results supported our previous study and suggest a target other than COX-2 for the antitumor effect of compound **B** (17). Since VEGF is a prerequisite for tumor invasion and its expression may be through either COX-2 dependent or independent pathway (20), the further studies are still deserved to validate VEGF as a target responsible for the conflicting results at the higher dose. 

**Table 1:** IC50 values (µM) for cytotoxic activity of compounds A and B towards MDA-MB-231 Cells.^a^

**Compounds **	**IC**_50 _ **(µM)**
**Celecoxib ** **A** **B**	7.45 (6.56-8.45) 17.95 (14.65-22.01) 9.2 (7.75-10.95)

^a^ Data are expressed as mean of three separate experiments run in triplicate and 95% confidence intervals

## Conclusion

In conclusion, our findings presented the first evidence of the ability of compounds** A** and **B **with tri-aryl structures resembling COX-2 inhibitors to prevent the tumor growth as well as metastasis to the lung against breast cancer. Our findings also suggest an underlying mechanism of the higher dose of compound **B** at which it was unable to inhibit the tumor growth. This occurrence may be attributed to the elevated level of VEGF expression through a COX-2 independent pathway. Overall, our data propose the use of compound **B** as a co-therapy in the protocols of cancer treatment; however, it needs further experiments to be validated. 

## References

[1] Torre LA, Bray F, Siegel RL, Ferlay J, Lortet-Tieulent J, Jemal A (2015). Global cancer statistics, 2012. CA Cancer J. Clin.

[2] Lee G, Walser TC, Dubinett SM (2009). Chronic inflammation, chronic obstructive pulmonary disease, and lung cancer. Curr.Opin. Pulm. Med.

[3] Jemal A, Bray F, Center MM, Ferlay J, Ward E, Forman D (2011). Global cancer statistics. CA Cancer J. Clin.

[4] Mousavi E, Tavakolfar S, Almasirad A, Kooshafar Z, Dehghani S, Afsharinasab A, Amanzadeh A, Shafiee S, Salimi M (2017). In-vitro and in-vivo assessments of two novel hydrazide compounds against breast cancer as well as mammary tumor cells. Cancer Chemother. Pharmacol.

[5] Holen I, Speirs V, Morrissey B, Blyth K (2017). In-vivo models in breast cancer research: progress, challenges and future directions. Dis. Model. Mech.

[6] Baliga MS, Meleth S, Katiyar SK (2005). Growth inhibitory and antimetastatic effect of green tea polyphenols on metastasis-specific mouse mammary carcinoma 4T1 cells in-vitro and in-vivo systems. Clin. Cancer Res.

[7] Smerage JB, Barlow WE, Hortobagyi GN, Winer EP, Leyland-Jones B, Srkalovic G, Srkalovic G, Tejwani S, Schott AF, O’Rourke MA, Lew DL, Doyle GV, Gralow JR, Livingston RB, Hayes DF (2014). Circulating tumor cells and response to chemotherapy in metastatic breast cancer: SWOG S0500. J. Clin. Oncol.

[8] Hajrezaie M, Paydar M, Looi CY, Moghadamtousi SZ, Hassandarvish P, Salga MS, Karimian H, Shams K, Zahedifard M, Abdul Majid N, Mohd Ali H, Abdulla MA (2015). Apoptotic effect of novel schiff based CdCl2 (C14H21N3O2) complex is mediated via activation of the mitochondrial pathway in colon cancer cells. Sci. Reports.

[9] Marsh S, McLeod HL (2007). Pharmacogenetics and oncology treatment for breast cancer. Expert Opin. Pharmacother..

[10] Jassem J (2007). The role of radiotherapy in lung cancer: where is the evidence? Radiother. Oncol..

[11] Duarte S, Carle G, Faneca H, Lima MC, Pierrefite-Carle V (2012). Suicide gene therapy in cancer: where do we stand now?. Cancer Lett..

[12] Shi S, Yao W, Xu J, Long J, Liu C, Yu X (2012). Combinational therapy: new hope for pancreatic cancer?. Cancer Lett..

[13] Chidambaram M, Manavalan R, Kathiresan K (2011). Nanotherapeutics to overcome conventional cancer chemotherapy limitations. J. Pharm. Pharm. Sci..

[14] Whittle JR, Lewis MT, Lindeman GJ, Visvader JE (2015). Patient-derived xenograft models of breast cancer and their predictive power. Breast Cancer Res..

[15] Thun MJ, Henley SJ, Patrono C (2002). Nonsteroidal anti-inflammatory drugs as anticancer agents: mechanistic, pharmacologic, and clinical issues. J. Natl. Cancer Inst.

[16] Jendrossek V (2013). Targeting apoptosis pathways by celecoxib in cancer. Cancer Lett.

[17] Norouzi M, Norouzi S, Amini M, Amanzadeh A, Irian S, Salimi M (2015). Apoptotic effects of two COX-2 inhibitors on breast adenocarcinoma cells through COX-2 independent pathway. J. Cell. Biochem.

[18] Miralinaghi P, Salimi M, Amirhamzeh A, Norouzi M, Kandelousi HM, Shafiee A, Amini M (2013). Synthesis, molecular docking study, and anticancer activity of triaryl-1, 2, 4-oxadiazole. Med. Chem. Res.

[19] Ramer R, Walther U, Borchert P, Laufer S, Linnebacher M, Hinz B (2013). Induction but not inhibition of COX-2 confers human lung cancer cell apoptosis by celecoxib. J. Lipid Res.

[20] Xu B, Wang Y, Yang J, Zhang Z, Zhang Y, Du H (2016). Celecoxib induces apoptosis but up-regulates VEGF via endoplasmic reticulum stress in human colorectal cancer in-vitro and in-vivo. Cancer Chemother. Pharmacol.

[21] Nie W, Ma X-l, Sang Y-x, Li Y-l, Gao X, Xu G-c, Shen GB, Shi HS, Liu XX, Wang FT, Wei YQ (2014). Synergic antitumor effect of SKLB1002 and local hyperthermia in 4T1 and CT26. Clin. Exp. Med.

[22] Zhang T, Li J, Dong Y, Zhai D, Lai L, Dai F, Deng H, Chen Y, Liu M, Yi Z (2012). Cucurbitacin E inhibits breast tumor metastasis by suppressing cell migration and invasion. Breast Cancer Res. Treat.

[23] Zheng T, Zhu Z, Wang Z, Homer RJ, Ma B, Riese RJ, Chapman HA Jr, Shapiro SD, Elias JA (2000). Inducible targeting of IL-13 to the adult lung causes matrix metalloproteinase–and cathepsin-dependent emphysema. J. Clin. Invest.

